# Psychosocial vascular interactions: It's a ‘shame’ we don't kNOw more

**DOI:** 10.1113/EP091104

**Published:** 2023-03-10

**Authors:** Tabitha V. Craig, Destiny R. Marston, Kurt J. Smith

**Affiliations:** ^1^ Cerebrovascular Health Exercise and Environmental Research Sciences Laboratory, School of Exercise Science, Physical and Health Education, Faculty of Education University of Victoria Victoria British Columbia Canada

**Keywords:** endothelial function, inflammation, nitric oxide, psychosocial stress, shame, stress, vascular health

Decreased endothelial function is associated with an increased risk for cardiovascular events. One of the primary mechanisms associated with decreased endothelial function is inflammation that inhibits nitric oxide (NO) vascular pathways (Hinterdobler et al., 2021). Factors such as age, high cholesterol and hypertension are well known to increase inflammation and reduced endothelial function, but it has recently been demonstrated that psychosocial stress is associated with both increased inflammation and peripheral endothelial dysfunction (Hinterdobler et al., 2021). Acute psychosocial stress (e.g., shame, embarrassment, anxiety) instigates the release of stress hormones and activates sympathetic nervous activity. Increased sympathetic nervous activity elevates circulating catecholamines, which alter the sensitivity of α‐ and β‐adrenergic receptors that can counter NO pathways involved in endothelium‐mediated relaxation (Hinterdobler et al., 2021). Increased pro‐inflammatory factors, such as cortisol, C‐reactive protein and interleukins, associated with higher stress hormone levels increase the risk of vascular dysfunction and disease (Hinterdobler et al., 2021; McGarity‐Shipley et al., 2022). Chronic psychosocial stress increases the risk of developing vascular dysfunction through prolonged exposure to circulating pro‐inflammatory factors and an increase in coping mechanisms (smoking, sedentary behaviour etc.) that exacerbate deleterious pro‐inflammatory mechanisms (Hinterdobler et al., 2021). Recently, it was demonstrated that acute psychosocial stress negatively impacts endothelial function. Whether this acute change in function is caused directly by an inflammatory cascade initiated by psychosocial stress or by an NO‐mediated factor remains to be seen (Clark et al., 2001).

McGarity‐Shipley et al. (2022) recently presented their study examining the effects of acute shame on endothelial function in young adults, published in the August 2022 issue of *Experimental Physiology*. The authors investigated brachial artery reactive hyperaemia to quantify endothelial function and the link between stress hormones and endothelial function following acute psychosocial distress. Using a pre/post crossover controlled experimental design, the authors exposed participants to a standardized 30 min shame protocol and observed transient impairments in brachial artery flow‐mediated vasodilatation (FMD). The authors reported higher cortisol levels on the day of the shame protocol, but did not observe a change in stress hormones or inflammatory biomarkers as a result of the shame protocol. Despite the absence of stress‐related inflammatory biomarkers, the reductions in FMD were interpreted as endothelium‐mediated vascular dysfunction attributable to an increase in psychosocial distress on the day. Although the lack of the hypothesized mechanistic explanation of endothelial dysfunction might be frustrating, McGarity‐Shipley et al. (2022) provide the first evidence demonstrating that acute psychological shame induced by psychosocial stress reduces peripheral endothelial function. This expands research by Clark et al. (2001) demonstrating that mental stress impairs vascular function, whereas humour increased endothelial function. Collectively, these studies provide a unique opportunity to engage in discourse related to mechanisms, methodology and future studies that might help to elucidate the links between mental health and vascular function.

McGarity‐Shipley et al. (2022) observed reductions in FMD in a shame protocol compared with control conditions when analysing the lowest FMD responses observed at 15 and 35 min post. This form of analysis was focused to account for individual variability in the time course of shame‐induced vascular dysfunction; however, it is hard to look past the fact that the time effect was not present when accounting for all measurements. Furthermore, despite higher cortisol levels during the shame trial, a lack of time effect indicated that the shame protocol itself did not result in significant alterations in inflammatory markers (cortisol or tumour necrosis factor‐α). Collectively, it is difficult to reconcile the mechanisms responsible for the reduction in FMD. Given that changes in FMD occurred independent of fluctuations in oral stress markers, it is probable that the reduction in endothelial function is driven by another mechanism or another inflammatory pathway. Nevertheless, the results are intriguing, and further exploration into the impact of psychosocial stressors on endothelial function is needed.

Stress‐induced excitation of the sympathetic nervous system can trigger the release of adrenaline and noradrenaline. Higher noradrenaline levels are associated with increased vasoconstriction, which is the likely culprit for the higher mean arterial pressure (81.9 ± 5.4 mmHg at baseline and 85.1 ± 5.5 mmHg at 15 min post) observed in the shame group compared with the control group in the study by McGarity‐Shipley et al. (2022). Adrenergically mediated vasoconstriction might trigger a counterbalanced response to FMD, as reviewed by Hinterdobler et al. (2021). Measurements of catecholamine levels are needed to rule this out as a possible mechanism in shame‐induced vascular dysfunction. Regardless, the elevated mean arterial pressure triggered by shame continued for ≤35 min after shame exposure. Chronically increased blood pressure is driven by the release of angiotensin II, which also increases the release of adhesion molecules from the endothelium. The interaction of elevated blood pressure and adhesion molecules results in the development of vascular disease over time (Hinterdobler et al., 2021). Studies that use adrenergic sympathetic blockade or modulation of blood pressure are needed to determine whether the acute impacts of shame on peripheral FMD are the result of autonomic activation rather pro‐inflammatory mediators alone.

In contrast to the detrimental influence of psychosocial stress on vascular function, there is evidence that positive social stressors might have a beneficial impact on vascular function. Sugawara et al. (2010), examined the effects of mirthful laughter on vascular function. They found that brachial artery FMD increased significantly after a 5 min period of laughter. They proposed two potential mechanisms behind the increased brachial artery FMD after laughter. The first is that laughter involves the contraction of multiple muscle groups, leading to increased cardiac output and peripheral blood flow, which are important for the mechanical stimulation pattern often theorized as being important for impacting endothelial function (Green & Smith, 2018). Another proposed mechanism is through laughter‐induced β‐endorphin release. Laughter and humour are associated with higher β‐endorphin levels (Berk et al., 1989). Stress hormones (i.e., β‐endorphins) released by the pituitary gland can upregulate nitric oxide synthase through the activation of $mu^{3}$ opiate receptors within endothelial cells (Stefano et al., 1995). Upregulation of nitric oxide synthase enhances nitric oxide production (Vanhoutte et al., 2017), which could potentially provide vascular health benefits (Green & Smith, 2018). Conversely, after negative psychosocial stress the $mu^{1}$ opioid receptor responds to β‐endorphin release by disturbing the balance between endothelin‐1 vasoconstriction and NO vasodilatory pathways (Wilbert‐Lampen et al., 2007). Thus, considering that an anticipatory effect of humour can raise β‐endorphins (Berk et al., 1989), although speculative, a potential anticipation before the shame protocol might have triggered an increase in β‐endorphins, resulting in disruption of vasoactive components (i.e., endothelin‐1 and NO) and, ultimately, reduced the FMD observed by McGarity‐Shipley et al. (2022). Analysis of circulating β‐endorphin and nitric oxide levels, while also investigating the impact of naloxone inhibition of β‐endorphins, in individuals experiencing both shame and mirthful laughter, would be a new and beneficial approach to reveal the mechanism involved in the psychosocial vascular dysfunction phenomenon.

McGarity‐Shipley et al. (2022) have provided insight into the effects of emotions on our peripheral vasculature and have laid out the groundwork for a multitude of investigations. Emotion‐induced changes to brachial artery FMD might produce a uniquely quantifiable physiological marker of mental health. Gaining further insight into how shame affects the vasculature might provide further characterization of the relationship between endothelial function and mental health. By examining the effects of shame‐related coping mechanisms on vascular function, unique insights into the complex relationship between psychosocial stress and physiological manifestations of this stress on vascular health are possible. McGarity‐Shipley et al. (2022) have left us in search of a mechanism to answer an intriguing question that could have implications for healthy vascular development in youth to adult transition and during healthy lifelong ageing. Further investigation into less obvious humoral factors caused by emotional distress that instigate inflammation and impairments to NO‐mediated vascular function are needed to quantify the mind–body connection.

## AUTHOR CONTRIBUTIONS

All authors have read and approved the final version of this manuscript and agree to be accountable for all aspects of thework in ensuring that questions related to the accuracy or integrity of any part of the work are appropriately investigated and resolved. All persons designated as authors qualify for authorship, and all thosewho qualify for authorship are listed.

## CONFLICT OF INTEREST

None declared.

## FUNDING INFORMATION

British Columbia Graduate Scholarship; National Sciences and Engineering Research Council (NSERC) of Canada.
